# Liquiritin ameliorates painful diabetic neuropathy in SD rats by inhibiting NLRP3-MMP-9-mediated reversal of aquaporin-4 polarity in the glymphatic system

**DOI:** 10.3389/fphar.2024.1436146

**Published:** 2024-09-04

**Authors:** Shuai-Ying Jia, Wen-Qin Yin, Wen-Mei Xu, Jiang Li, Wei Yan, Jing-Yan Lin

**Affiliations:** ^1^ Department of Anesthesiology, The Affiliated Hospital of North Sichuan Medical College, Nanchong, China; ^2^ Department of Medical Imaging, The Affiliated Hospital of North Sichuan Medical College, Nanchong, China

**Keywords:** painful diabetic neuropathy, glymphatic system, aquaporin-4, liquiritin, matrix metalloproteinase-9

## Abstract

**Background:**

Despite advancements in diabetes treatment, the management of Painful Diabetic Neuropathy (PDN) remains challenging. Our previous research indicated a significant correlation between the expression and distribution of Aquaporin-4 (AQP4) in the spinal glymphatic system and PDN. However, the potential role and mechanism of liquiritin in PDN treatment remain uncertain.

**Methods:**

This study established a rat model of PDN using a combination of low-dose Streptozotocin (STZ) and a high-fat, high-sugar diet. Rats were treated with liquiritin and MCC950 (an NLRP3 inhibitor). We monitored fasting blood glucose, body weight, and mechanical allodynia periodically. The glymphatic system’s clearance function was evaluated using Magnetic Resonance Imaging (MRI), and changes in proteins including NLRP3, MMP-9, and AQP4 were detected through immunofluorescence and Western blot techniques.

**Results:**

The rats with painful diabetic neuropathy (PDN) demonstrated several physiological changes, including heightened mechanical allodynia, compromised clearance function within the spinal glymphatic system, altered distribution of AQP4, increased count of activated astrocytes, elevated expression levels of NLRP3 and MMP-9, and decreased expression of AQP4. However, following treatment with liquiritin and MCC950, these rats exhibited notable improvements.

**Conclusion:**

Liquiritin may promote the restoration of AQP4 polarity by inhibiting NLRP3 and MMP-9, thereby enhancing the clearance functions of the spinal cord glymphatic system in PDN rats, alleviating the progression of PDN.

## 1 Introduction

Diabetes, as a long-term chronic disease, is accompanied by various severe complications. According to data from 2021, the global prevalence of diabetes among people aged 20 to 79 is estimated to be 10.5%, equating to approximately 536.6 million individuals (Sun et al., 2022). About 20%–30% of these patients are likely to develop Painful Diabetic Neuropathy (PDN) (Abbott et al., 2011; Aronson et al., 2021). PDN is characterized by severe foot pain and electric shock sensation, causing prolonged suffering for patients. Concurrently, individuals with PDN often report a decline in quality of life, anxiety, depression, and sleep disturbances (Gylfadottir et al., 2020). Despite extensive research, the fundamental causes of diabetic peripheral neuropathy have not been fully elucidated. Currently, the treatment of PDN primarily relies on pharmacotherapy. As recommended by the International Consensus Group on Diabetic Neuropathy, Tricyclic Antidepressants (TCAs), Duloxetine, Pregabalin, and Gabapentin are considered first-line treatment options (Tesfaye et al., 2011). However, the use of these medications may be associated with various side effects, including nausea, drowsiness, and constipation (Griebeler et al., 2014). Treating PDN solely with these drugs could result in patient dissatisfaction with the treatment outcomes (Tesfaye et al., 2022). Consequently, it is essential to delve into the pathogenesis of PDN and explore alternative effective pharmacological treatment strategies to enhance clinical outcomes.

Although the pathogenesis of PDN has not been fully elucidated, the release of pro-inflammatory factors and metabolic abnormalities are considered to play a key role in the onset and progression of PDN (Zychowska et al., 2013; Talbot et al., 2010). It is particularly noteworthy that the Central Nervous System (CNS) lacks a lymphatic system to assist in the clearance of pro-inflammatory factors (Jessen et al., 2015). Recent studies have discovered that the glymphatic system plays a significant role in the waste metabolism of the CNS (Iliff et al., 2013b). Aquaporin-4 (AQP4), densely expressed in the end-feet of astrocytes (i.e., polarity distribution), is primarily responsible for the metabolic functions of the glymphatic system (Mestre et al., 2018). Similar structures have also been found in the spinal cord (Liu et al., 2018). AQP4 is widely distributed in the central nervous system, facilitating rapid transport of water and metabolic waste. The polarized distribution of AQP4 forms the structural basis for maintaining normal clearance functions of the glymphatic system (Yin et al., 2019). Under physiological conditions, cerebrospinal fluid (CSF) enters the brain parenchyma from the perivascular spaces (Virchow-Robin spaces, VRS) adjacent to arteries, mixing with interstitial fluid (ISF). CSF-ISF then flows along the perivenous VRS into the bloodstream or cervical lymphatic system. Similarly, ISF and waste products within the brain parenchyma are cleared via AQP4-dependent glymphatic pathways or perivascular spaces (Jessen et al., 2015). Moreover, AQP4 plays a crucial role in maintaining extracellular space volume, water homeostasis, and glutamate balance in the central nervous system (Haj-Yasein et al., 2012; Seifert et al., 2006; Das et al., 2012). Studies indicate that inhibition of AQP4 reduces the clearance capacity of the glymphatic system, underscoring the importance of normal AQP4 expression for this function (Gomolka et al., 2023). Research also shows polarized distribution of AQP4 in the cortical regions of healthy individuals, whereas in Alzheimer’s disease, AQP4 polarization is disrupted (AQP4 depolarization) (Zeppenfeld et al., 2017). Similarly, spinal cord slices from amyotrophic lateral sclerosis (ALS) and multiple sclerosis (MS) rodent models exhibit similar depolarization patterns (Dai et al., 2017; Wolburg-Buchholz et al., 2009). In our previous research, we observed a polarity change in AQP4 within the glymphatic system of the spinal cord in rats with PDN (Wang G. Q. et al., 2022). Moreover, we found that β-hydroxybutyrate and metformin alleviate PDN symptoms by restoring the polarity of AQP4 in the spinal glymphatic system (Wang F. X. et al., 2022; Xu et al., 2023). Therefore, further study into the distribution and regulatory mechanisms of AQP4 in the glymphatic system is of significant importance for finding effective PDN treatments.

The NOD-, LRR- and pyrin domain-containing protein 3 (NLRP3) inflammasome, a key member of the NOD-like receptor family, consists of NLRP3, Apoptosis-associated Speck-like protein containing a CARD, and pro-Casp-1 protein (Sun et al., 2019). Recent studies have identified dysfunction of the NLRP3 inflammasome as one of the mechanisms in the development of chronic pain (Starobova et al., 2020; Ren et al., 2021). Increasing evidence suggests that inhibiting the activation of the NLRP3 inflammasome can alleviate symptoms of PDN (Zhang et al., 2022; Zheng T. et al., 2021). Additionally, the expression of the NLRP3 inflammasome is closely associated with the number of astrocytes (Luo et al., 2019). Inhibition of the NLRP3 inflammasome plays a role in reducing brain edema after cerebral ischemia-reperfusion and also affects the distribution of AQP4 (Wang et al., 2020). Matrix Metalloproteinase-9 (MMP-9) has an anchoring role in the polar distribution of AQP4, and its increased expression level is related to the change in polarity of AQP4 in mice (Zheng R. et al., 2021; Wang et al., 2021). The NLRP3 inflammasome may be involved in regulating the expression of MMP-9 (Yamaguchi et al., 2023; Peng et al., 2020). Therefore, modulating NLRP3 to inhibit the overexpression of MMP-9 or correct the abnormal distribution of AQP4 could be a potential strategy for treating PDN.

Liquiritin, a flavonoid extracted from licorice, is one of the main constituents of this plant (Cheng et al., 2021). It demonstrates significant potential in preventing inflammation (Yang et al., 2021), alleviating pain (Chen et al., 2022), anti-cancer (Wang Q. et al., 2022), and treating coughs (Zhang et al., 2021) and allergic reactions (Wang L. et al., 2023). Furthermore, studies suggest that liquiritin is an effective anti-inflammatory and anti-damage drug. Its mechanism involves the downregulation of pro-inflammatory cytokines such as Tumor Necrosis Factor-alpha (TNF-α) and interleukins (IL-6, IL-1β), while upregulating the anti-inflammatory cytokine IL-10 (Li et al., 2018). This action is not only significant in the treatment of neuropathic pain but also inhibits the activation of spinal astrocytes caused by pain (Zhang M. T. et al., 2017).

However, despite the extensive research on liquiritin in the prevention of inflammation and pain treatment, the efficacy and potential mechanisms of liquiritin in treating PDN remain largely unclear. Therefore, this study aims to investigate the impact of liquiritin on the functional aspects of the spinal cord glial lymphatic system and the expression sites of the AQP4 protein in PDN rats. Additionally, we will examine whether NLRP3 and MMP-9 are involved in this process.

## 2 Materials and methods

### 2.1 Animals

Eighty adult male Sprague-Dawley rats (body weight: 160–180 g, age: 3–8 weeks) were acquired from the Experimental Animal Farm of Sichuan Province. These rats were housed individually in the Laboratory Animal Facility at North Sichuan Medical College, maintained at a temperature of 24°C ± 1°C and a relative humidity of 55% ± 5%. They had *ad libitum* access to food and water, under a 12-h light/dark cycle (lights on at 8:00 a.m. and off at 8:00 p.m.). The Institutional Ethics Committee of North Sichuan Medical College approved all experimental protocols (authorization number: 2023059). All procedures adhered strictly to the ARRIVE guidelines for the Care and Use of Experimental Animals.

### 2.2 Establishment and grouping of PDN rat models

**FIGURE 1:**
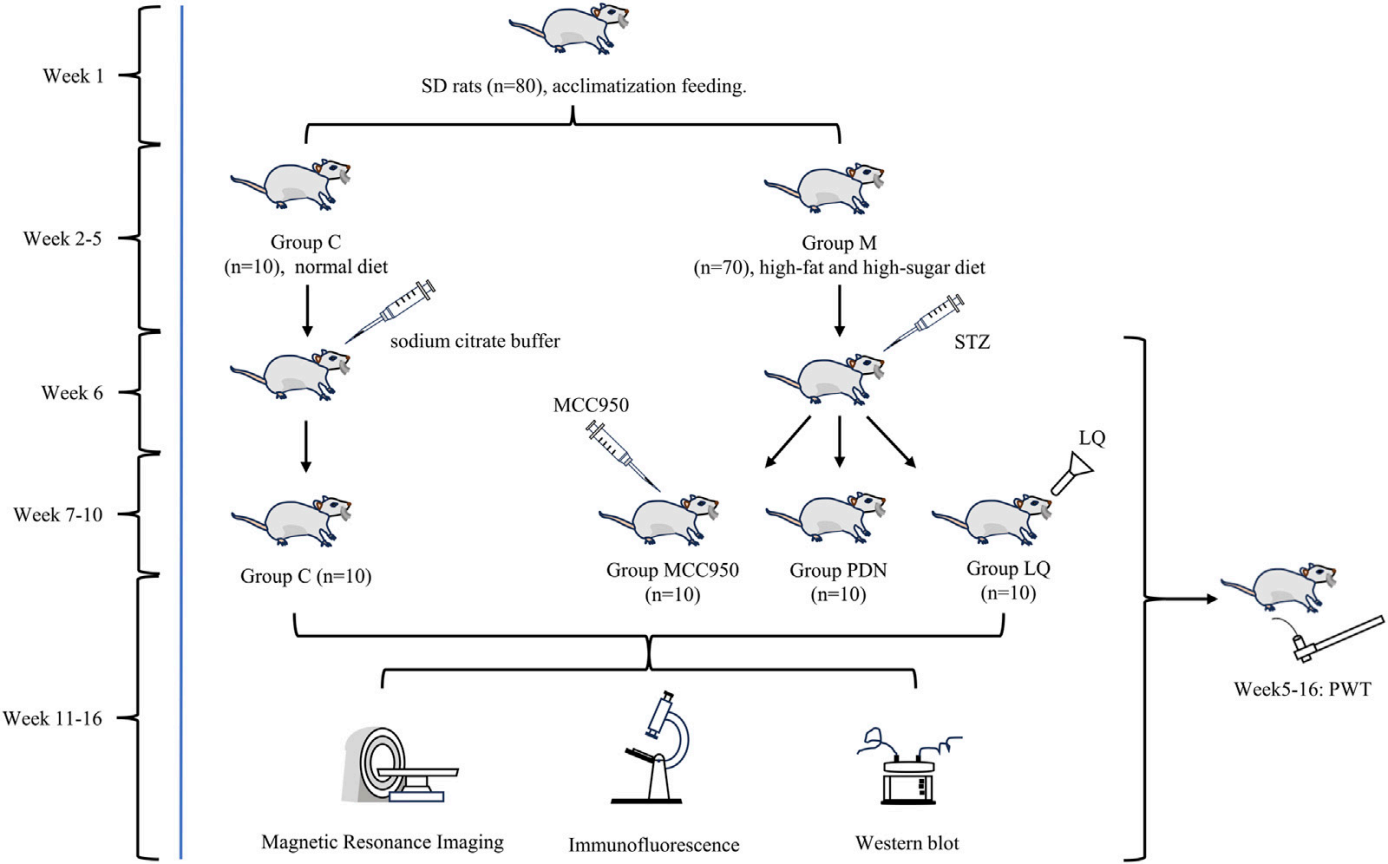
Experimental flow chart. The group LQ was administered liquiritin orally each day, while the group MCC950 received daily intraperitoneal injections of MCC950. The group C and group PDN rats were respectively administered gastric gavage and intraperitoneal injections of saline solution. A month after treatment, 4 rats from each group underwent MRI examinations. One week after the examination, spinal cords were extracted for Western Blot (WB) analysis, and the remaining 6 rats underwent immunofluorescence detection.

In the 16-week experiment, rats were initially randomized into a control group (group C, n = 10) and a model group (group M, n = 70). During the first week, both groups underwent adaptive feeding. Starting from the second week, the group C was maintained on a normal diet, while the group M switched to a high-fat, high-sugar diet (including 10% sucrose, 10% lard, and 5% cholesterol, purchased from Xiaoshuyoutai (Beijing) Biotechnology Co., LTD., catalog number D12450J), with weekly measurements of body weight and blood glucose. Starting from the 6th week, a single intraperitoneal injection of STZ (35 mg/kg, 1.0 mL/kg, HY-13753, MedChemExpress, Monmouth, NJ, United States) dissolved in 10 mmol/L citrate buffer (pH 4.5) was administered to induce diabetes (Furman, 2021). From 11 p.m. the night before the injection to 8 a.m. the next day, all rats were fasted with free access to water. After the completion of STZ injections, the rats in Group M returned to their original high-fat, high-sugar feeding regimen. 24 h later, blood samples were collected from the tail vein to measure fasting blood glucose levels. A fasting blood glucose level above 16.7 mmol/L indicated successful diabetes induction, this indicator has been employed in numerous diabetes-related studies (Wang G. Q. et al., 2022; Wang F. X. et al., 2022; Xu et al., 2023; Li et al., 2024). Rats in the group C were injected with an equivalent volume of sodium citrate buffer. Concurrently, Paw Withdrawal Threshold (PWT) was measured weekly. After another month of feeding, rats from the group M displaying PDN symptoms such as polydipsia, polyphagia, polyuria, weight loss, elevated fasting blood glucose, and reduced PWT were selected, these unselected rats were humanely euthanized following ethical guidelines. The selected 30 PDN rats were randomly divided into a group PDN, an NLRP3 inhibitor group (group MCC950), and a liquiritin treatment group (group LQ), with 10 rats in each group for follow-up experiments. All animals were anesthetized with 3.5% sevoflurane before sacrifice. This modeling process has been validated in preliminary studies by our group and confirmed in other studies. The experimental design is illustrated in Figure 1.

### 2.3 Drug preparation and administration

Group LQ: All rats underwent treatment with LQ (AB0619, Alfa Biology, Chengdu, China), which was first dissolved in saline and administered via gastric gavage at a dosage of 20 mg/kg. The gavage was administered each morning from 8:00 to 10:00 a.m., continuously for 30 days.

Group MCC950: All rats underwent treatment with MCC950 (HY-12815, MedChemExpress, Monmouth, NJ, United States), which was similarly dissolved in saline and administered at a dose of 6 mg/kg via intraperitoneal injection. The injections were carried out once every 2 days, totaling 15 injections throughout the experiment.

Group PDN: Rats in the control group received an equivalent volume of saline administered using the same methods and frequency of intraperitoneal injection and gastric gavage.

### 2.4 Paw withdrawal threshold (PWT) detection

We chose PWT as a common and classic tool to analyze neuropathic pain status (Nirogi et al., 2012). In the 6th week of the experiment, a designated experimenter began assessing the PWT of the rat’s hind paws using the Von Frey filament kit (US PAT. 58239698512259, North Coast, CA, United States), with assessments conducted once every 7 days, between 8:00 a.m. and 11:00 a.m. The evaluation method involved vertically pressing Von Frey filaments to the central position of the rat’s hind foot. A positive withdrawal response was indicated when the rat displayed behaviors such as retracting its foot or licking its paw (Chaplan et al., 1994). If three out of five consecutive experiments resulted in a positive withdrawal response, the value of that specific Von Frey filament was defined as the PWT. Specific steps included placing each rat on a mesh metal plate, allowing its feet to relax, and conducting the test after a 15 min period of environmental acclimatization (Su et al., 2019). Stimulation began with low-intensity Von Frey filaments. If the withdrawal response was negative, an experiment with an adjacent higher intensity filament was conducted until a positive withdrawal response was achieved. Five different points were tested on each rat, with each press lasting 5 s and an interval of approximately 30 s between presses. To prevent foot injury and in accordance with instrument specifications, the type of Von Frey filament used did not exceed a maximum of 26 g (Aman et al., 2016; Tokhi et al., 2023).

### 2.5 Magnetic resonance imaging (MRI) measurement

Following the treatment cycle, four rats from each group were selected for MRI scanning. These MRI scans were conducted in the MRI Room at the Affiliated Hospital of North Sichuan Medical College, utilizing a 3.0T clinical MR system (Discovery MR750, GE, United States). After the rats were anesthetized with sevoflurane, they were positioned in an eight-channel, rat-specific coil, and anesthesia was maintained with sevoflurane (2.5%–3.0%) combined with oxygen (2.5 L/min) for the duration of the experiment. The scanning region was centered on the rat’s 13th thoracic vertebra, extending 2.5 cm above and below this point. A rapid spin echo sequence T1-weighted MRI scan was employed to observe contrast agent movement into and out of the spinal cord. The scanning parameters were set as follows: TR (Time of Repetition): 450.0 ms, TE (Time to Echo): 5.4 ms, slice thickness: 2.0 mm, slice space: 1.0 mm, Field of View (FOV): 6.5 cm × 6.5 cm, matrix size: 256 × 256, flip angle: 90°, bandwidth: 31.25 kHz, and frequency direction: Anterior/Posterior (A/P) (Wang F. X. et al., 2022; Xu et al., 2023). After obtaining the initial image, the rats were positioned prone on the operating table, with elevation under the lower abdomen, and a contrast agent was injected into the subarachnoid space using a 50 μL syringe (Gaoge, China).

Through repeated experiments, we found that 10% Gadopentetic acid (Gd-DTPA) had an excellent enhancement effect (100 µL Gd-DTPA diluted in 900 µL sterile saline). The L6 vertebra was used as a positioning marker, a successful puncture was indicated by a rapid lateral movement of the rat’s tail (Thomas et al., 2016). We injected 25 µL of the diluted Gd-DTPA into the L4/5 subarachnoid space at a consistent rate over 5 min. Following the injection, the needle was retained in place for an additional 3 min to prevent backflow. Subsequently, the rat was transferred to the coil for MRI scanning after needle removal.

Images were acquired at intervals of 1, 15, 30 min, and 1, 1.5, 2, 2.5, 3, and 6 h post Gd-DTPA injection. Throughout the experiment, rats’ vital signs were continuously monitored; heart rate was maintained between 250 and 450 beats/min, and body temperature was kept within 36.5°C–37.5°C using a feedback-controlled air heating blower to keep warm (Rapid Electric, Brewster, NY, United States). Between anesthesia sessions, animals were allowed to drink freely upon regaining consciousness. At each time point, the largest cross-sectional image of the spinal cord was identified, and the images of the upper and lower levels to it were also selected for analysis. Two blinded evaluators used the RadiAnt DICOM Viewer (64 bit; Version 2021.1, Medixant, Poland) to measure MRI Signal Intensity (MRI SI) in the gray matter of the spinal cord. The average of three image measurements was taken as the final MRI SI for each rat. After initial trials, we decided to focus MRI SI measurements on a consistent area of interest, approximately 0.04 cm^2^ in size, encompassing most of the spinal cord’s gray matter, ensuring an error margin not exceeding 300.

### 2.6 Western blot (WB)

Samples were placed into a 2 mL grinding tube, followed by the addition of two 3 mm grinding beads and RIPA lysis buffer (Beyotime Biotechnology, Shanghai, China), and then subjected to a high-speed low-temperature tissue grinder (temperature: −20°C, grinding 4 times, each for 60 s). Subsequently, the samples were removed and lysed in a refrigerator at 4°C for 30 min, followed by centrifugation (4°C, 12,000 rpm, 10 min). After centrifugation, the supernatant was collected, and the protein concentration was determined using a BCA protein assay kit (Beyotime Biotechnology, Shanghai, China), The samples, after electrophoresis, were transferred onto PVDF membranes. The PVDF membranes were blocked in 5% skim milk diluted with TBST Buffer for 2 h, followed by incubation with primary antibodies (antibody concentrations: AQP4 (Proteintech, AB_2827426, 1:1,000); MMP-9 (Servicebio, AB_10796269, 1:1,000); NLRP3 (Huabio, AB_3069980, 1:1,000); α-Tubulin (Proteintech, AB_11042766, 1:50,000)) at 4 C overnight. After three washes, the PVDF membranes were incubated with secondary antibodies (dilution concentration: 1:5,000) at room temperature for 2 h. The integral optical density (IOD) of the target proteins was measured using Tanon Fluorescence Image Analysis System Software V2.0.

### 2.7 Immunofluorescence

Six rats in each group were randomly selected to undergo transcardiac perfusion after deep anesthesia with phosphate buffered saline and 4% paraformaldehyde, respectively. Subsequently, the L4–L6 segments of the spinal cord were excised and preserved in 4% paraformaldehyde. 48 h later, according to the experimental protocol, these tissues were embedded in paraffin blocks. Subsequently, 4 µm thick horizontal sections were prepared.

Paraffin sections were initially dewaxed, followed by antigen retrieval and endogenous peroxidase blocking. Subsequently, the sections were blocked with bovine serum albumin at room temperature for at least 30 min. A mixture of AQP4 antibody (Rabbit/IgG, Proteintech, AB_2827426, 1:100), GFAP antibody (Mouse/IgG3, Proteintech, AB_10838694, 1:200), and anti-CD31 antibody (Rabbit/IgG, Abcam, AB_11218334, 1:100) was added and incubated overnight at 4°C. After washing, secondary antibodies Cy3-labeled goat anti-rabbit (Servicebio, GB21303) and CY3-labeled goat anti-mouse (Servicebio, GB21301) were added, and the sections were incubated with DAPI for 10 min. Following PBS washing, the sections were sealed with anti-fluorescence quenching sealant.

The sample was subsequently mounted and examined utilizing an Olympus FV1200 confocal laser scanning microscope equipped with a 40× objective lens. Specific parameters were configured as follows: DAPI - HV 477, Gain 2, Offset 22; Alexa Fluor 488 - HV 555, Gain 1, Offset 59; Alexa Fluor 647 - HV 466, Gain 1, Offset 38. Representative images were selected to depict the polarity reversal of the AQP4 protein. Laser power, pinhole size, and image detection parameters were consistently upheld to ensure impartial representation across all samples.

### 2.8 Quantification of AQP4 polarization and quantification of GFAP fluorescence intensity

To assess the activation of astrocytes and changes in AQP4 polarity, we utilized GFAP + AQP4 and CD31 +AQP4 dual immunofluorescence staining techniques, selecting five slices randomly from each group for analysis.

#### 2.8.1 GFAP fluorescence intensity

Initially, we defined Regions of Interest (ROI) on each image, ensuring consistent ROI selection criteria across all groups. Measurements were conducted in the central canal, ventral horn, and dorsal horn regions. Subsequently, using ImageJ version 1.54f bundled with Java 1.8.0_322 software (National Institutes of Health), we isolated the GFAP signal channel and removed background fluorescence to measure the GFAP immunofluorescence intensity within each ROI, calculating averages to obtain the GFAP immunofluorescence intensity for each image. Finally, after compiling all image data, we utilized GraphPad Prism statistical software to compare the differences in GFAP expression among the three groups.

#### 2.8.2 Quantification of AQP4 polarization

AQP4 is primarily localized around the vascular vicinity of the astrocytic end-feet, a distribution we refer to as “AQP4 Polarization.” To better quantify this “polarization,” using AQP4 + CD31 images as examples, our measurement methodology was consistent with previous studies (Kress et al., 2014). In ImageJ software, we isolated the AQP4 signal channel and selected perivascular areas in the central canal, ventral horn, and dorsal horn regions of each image for measurement, calculating their average AQP4 immunofluorescence intensity. Subsequently, through threshold analysis, we measured the percentage of the total image area where AQP4 immunofluorescence intensity was less than that around blood vessels, defining this ratio as “AQP4 polarization.” Similar methods were employed in GFAP + AQP4 fluorescence images to quantify AQP4 polarity.

### 2.9 Statistical analysis

Statistical analyses were conducted using GraphPad Prism 8. A significance threshold of 0.05 was applied to all tests. Data are presented as mean ± SEM. One-way analysis of variance (ANOVA) was employed for comparing group means, while repeated-measures ANOVA was utilized for assessing differences in PWT and MRI signal intensity. Post-hoc analyses were performed using the Bonferroni correction method.

## 3 Results

### 3.1 Metabolic characteristics of rats in each group

**FIGURE 2:**
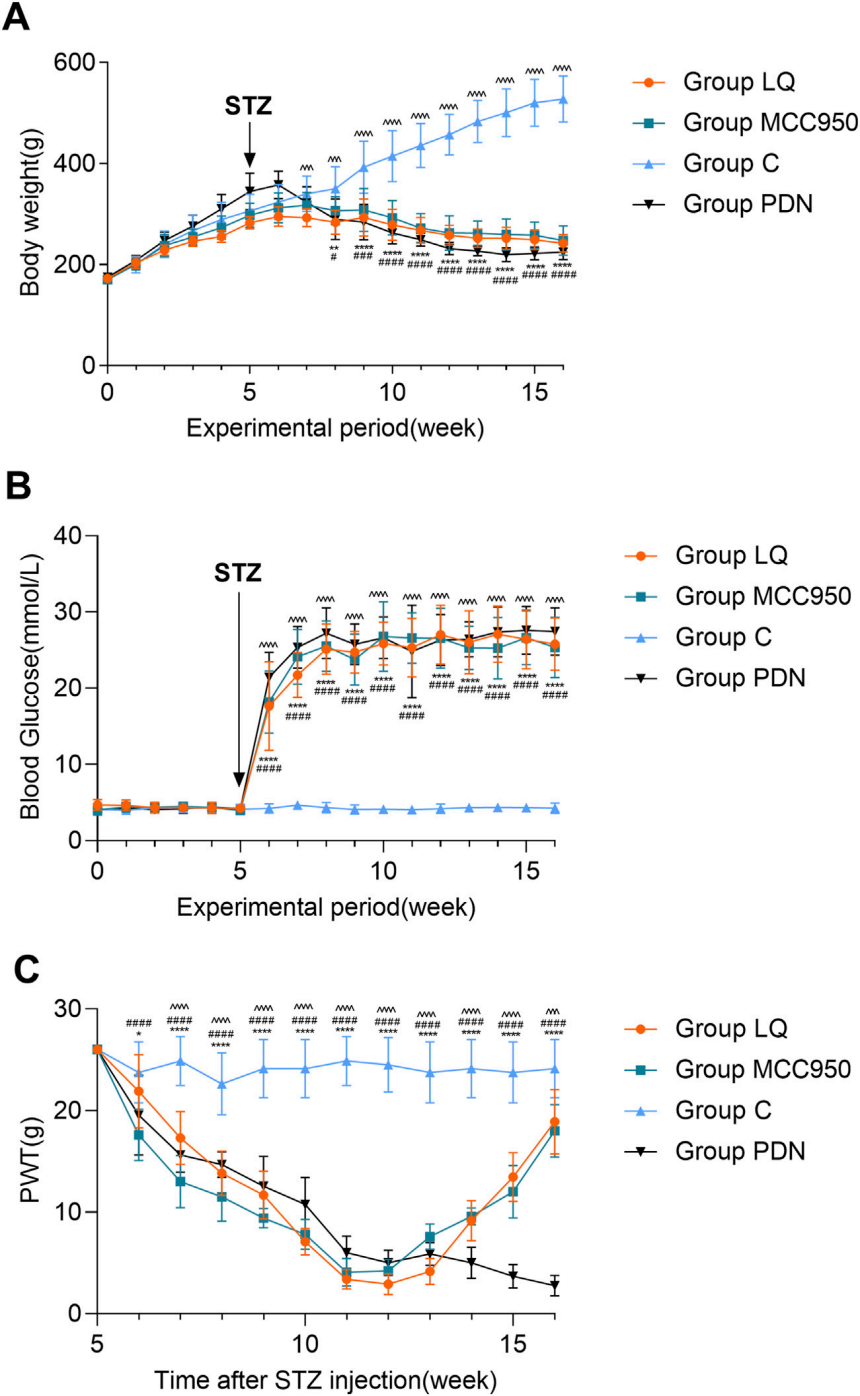
The trend of body weight, blood glucose and PWT change in rats. **(A)** The trend of body weight in Group C, Group PDN, Group LQ and Group MCC950. **(B)** The trend of blood glucose in Group C, Group PDN, Group LQ and Group MCC950. **(C)** The trend of PWT in Group C, Group PDN, Group LQ and Group MCC950. The one-way analysis of variance (ANOVA) was used to compare means between groups. The repeated-measures ANOVA was used to compare the PWT. Post hoc analyses were conducted using the Bonferroni correction method. Refer to Figure 1 for the meaning of the grouping abbreviation. Values were presented as mean ± SEM. Group C compared with group PDN, ^*^ *p* < 0.05, ^**^ *p* < 0.01, ^****^ *p* < 0.0001; Group C compared with group MCC950, ^#^ *p* < 0.05, ^###^ *p* < 0.001, ^####^ *p* < 0.0001; Group C compared with group LQ, ^^^^^ *p* < 0.001, ^^^^^^ *p* < 0.0001.

Figure 2A indicates that throughout the experimental period, the body weight of the rats in group C exhibited a continuous upward trend. In contrast, the rats in the other three groups (LQ, MCC950, and PDN) showed an initial increase in body weight followed by a decline. Specifically, beginning from the 7th week of the experiment, the body weight of the rats in the group LQ was significantly lower than that of the group C (*p* < 0.05). Similarly, the rats in the group MCC950 and group PDN showed a significant reduction in body weight compared to the group C starting from the 8th week (*p* < 0.05). Figure 2B further reveals another key observation in the experiment. After intraperitoneal injection of STZ, the blood glucose levels in the PDN, LQ, and MCC950 groups were significantly higher than those in the group C (*p* < 0.05), and this hyperglycemic state remained stable thereafter. In contrast, the blood glucose levels in the group C rats consistently remained within the normal range. Additionally, except for the group C, the rats in the other three groups exhibited typical symptoms of hyperglycemia, such as increased drinking, overeating, and polyuria. These observations indicate the successful establishment of a diabetes model. In the Figure 2C, a significant decrease in PWT was observed in the three experimental groups compared to the group C starting from the 7th week (*p* < 0.05). Subsequently, the PWT continued to decline in the group PDN, while in the MCC950 and LQ groups, there was a gradual increase in PWT during the 11th and 12th weeks following pharmacological intervention. This indicates that both LQ and MCC950 are effective in alleviating mechanical allodynia. However, blood glucose levels remained elevated in the LQ and MCC950 groups of rats. This implies that while LQ and MCC950 alleviate the symptoms of PDN, they do not affect blood glucose levels.

### 3.2 LQ and NLRP3 inhibitor improved the metabolic rate of Gd-DTPA in rat spinal glymphatic system

**FIGURE 3:**
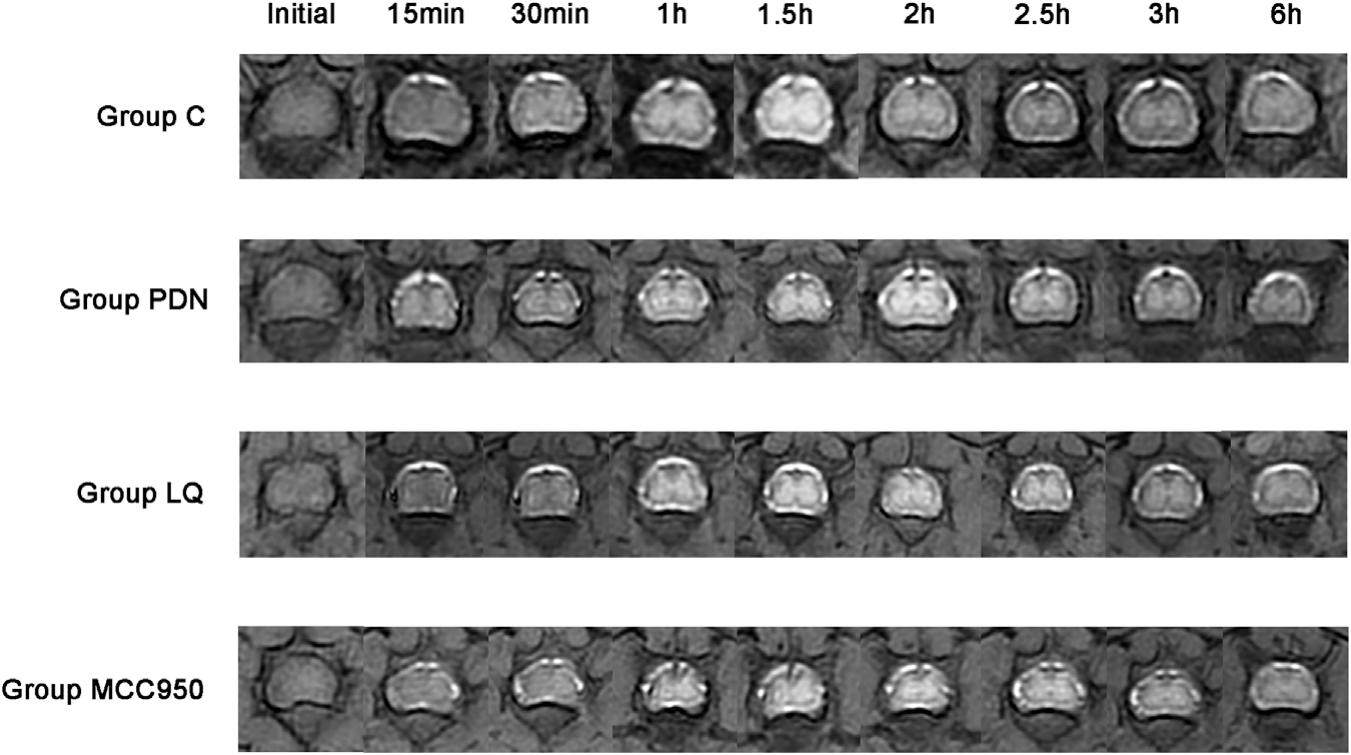
Metabolic image of Gd-DTPA at spinal cord lumbar enlargement in rats (Initial–6 h). Initial: Before injection of Gd-DTPA; 15 min: Inject Gd-DTPA for 15 min; 30 min: Inject Gd-DTPA for 30 min; 1 h: Inject Gd-DTPA for 1 h; 1.5 h: Inject Gd-DTPA for 1.5 h; 2 h: Inject Gd-DTPA for 2 h; 2.5 h: Inject Gd-DTPA for 2.5 h; 3 h: Inject Gd-DTPA for 3 h; 6 h: Inject Gd-DTPA for 6 h. Refer to Figure 1 for the meaning of the grouping abbreviation.

In our study, we employed MRI to observe the metabolic activity of Gd-DTPA contrast agents in the lumbar enlargement of the rat spinal cord, aiming to assess the metabolic function of the spinal glymphatic system. Following the injection of the contrast agent into the subarachnoid space, an initial stage manifests as a discontinuous distribution, subsequently progressing into the spinal cord gray matter. When the absorption reaches its peak, the characteristic “butterfly sign” is formed. According to the results in Figure 3, it is evident that, compared to the other three groups, rats in the group PDN exhibited a delayed onset and slower disappearance of the high-intensity “butterfly sign.” Concurrently, compared to the other three groups, the PDN group requires a longer time to reach peak signal intensity and exhibits a slower decline. Additionally, to facilitate data comparison, we tabulated all the data in detail in Table 1. Analysis of Table 1 revealed that the rate of SI change in the remaining three groups of rats was statistically significant compared to the group PDN (*p* < 0.01). This indicates that the metabolic function of the glymphatic system in the PDN rats was impaired, but improved and recovered following treatment with LQ and MCC950.

**TABLE 1:** Detailed MRI signal intensity obtained from the lumbar enlargement.

	Group C (n = 4)	Group PDN (n = 4)	Group LQ (n = 4)	Group MCC950 (n = 4)	*p-*value
Initial	5,871 ± 471.64	5,475 ± 300.79	6,256 ± 91.86	5,724 ± 555.78	0.58
PEAK SI	8,399 ± 643.53	8,323 ± 261.68	8,978 ± 138.29	8,493 ± 718.37	0.8
SIX SI	6,222 ± 568.74	7,234 ± 214.40	6,772 ± 189.62	6,179 ± 621.80	0.33
PEAK TIME	1.63 ± 0.13	2.13 ± 0.13	1.75 ± 0.14	2 ± 0.20	0.14
SIPH	498 ± 24.79^**^	280 ± 13.54	523 ± 43.60^**^	580 ± 30.05^**^	<0.01

Initial: the initial SI. PEAK SI: the peak of SI. SIX SI, SI values six hours post-injection. PEAK TIME: the time to peak SI. SIPH: the rate of change in SI, representing the rate at which the signal intensity decreases from PEAK SI to SIX SI. The calculation formula is SIPH = (Mean_(PEAK SI)_- Mean_(SIX SI)_)/(6- Mean_(PEAK TIME)_). Refer to Figure 1 for the meaning of the grouping abbreviation. Values were presented as mean ± SEM. Compared with group PDN.

^**^ *p* < 0.01.

### 3.3 LQ and MC950 can inhibit astrocyte activation and restore the polarity distribution of AQP4 around astrocytes

**FIGURE 4:**
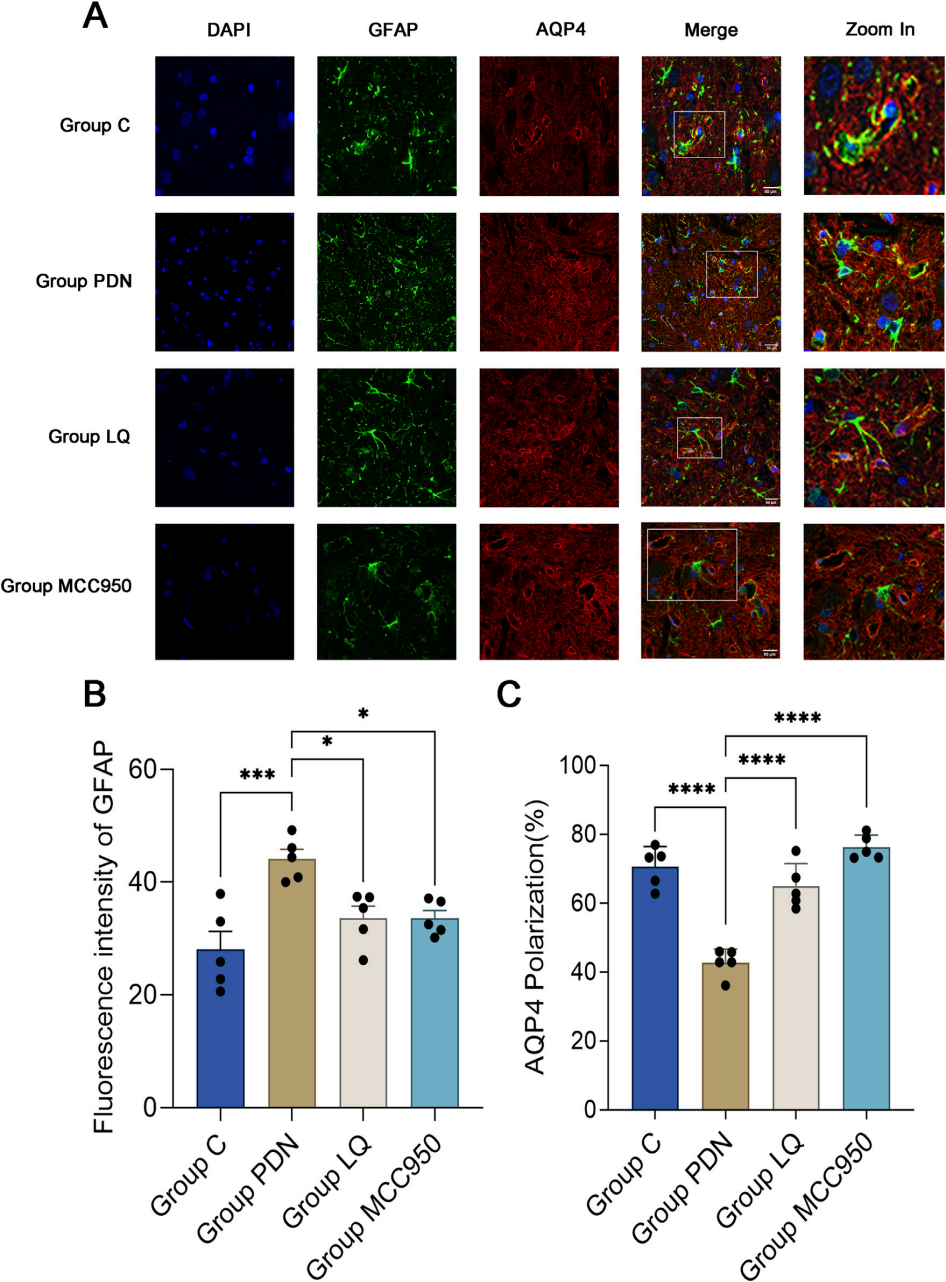
Immunofluorescence results of AQP4 and GFAP. **(A)** Immunofluorescent staining was performed on the lumbar enlargement segments of the spinal cord in the C, PDN, LQ, and MCC950 groups of rats. Activated astrocytes were marked with GFAP and identified by green fluorescence. AQP4 protein was identified with red fluorescence, and cell nuclei were counterstained with DAPI (blue). Enlarged sections detailed the polarity reversal of AQP4 protein. Scale bar = 50 µm. **(B)** Quantitative analysis of fluorescence in activated astrocytes across the groups. **(C)** Quantitative analysis of fluorescence polarization of AQP4. Refer to Figure 1 for the meaning of the grouping abbreviation. Values were presented as mean ± SEM. Compared to the group PDN, ^*^ *p* < 0.05, ^***^ *p* < 0.001, ^****^ *p* < 0.0001.

This study employed specific markers for Glial Fibrillary Acidic Protein (GFAP) to precisely label astrocytes, aiming to reveal changes in the localization and expression levels of AQP4 around activated astrocytes. Red fluorescence represents AQP4, and green should represent GFAP. In Figure 4A, we observed a dense distribution of AQP4 around astrocytes in group C, corresponding to the previously described “polarized” state. In contrast, in the spinal cord tissue of rats in the group PDN, AQP4 distribution around astrocytes was reduced and more dispersed, with irregular fluorescence localization, indicative of a “depolarized” distribution state. Following treatment with LQ and MCC950, fluorescence localization indicated an increase and concentration of AQP4 distribution around astrocytes, restoring the “polarized” state. Quantitative analysis shown in Figure 4B indicates that the GFAP fluorescence intensity in the group PDN was significantly higher than that in the group C (group PDN: 44.12 ± 1.52; group C: 28.06 ± 2.90; *p* < 0.001), and also higher than in the LQ and MCC950 treatment groups (group LQ: 33.60 ± 1.90, group MCC950: 33.58 ± 1.24, *p* < 0.05 for both). Additionally, we utilized ImageJ software to capture the fluorescence intensity of AQP4 and GFAP. This intensity was used to quantify the expression of AQP4 around astrocytes, in order to analyze whether AQP4 exhibits a “polarized” distribution. The results are shown in Figure 4C. The expression of AQP4 around astrocytes in PDN group rats decreased, indicating the disappearance of its polar distribution (the “polarization” ratio of AQP4 in C group rats was 70.65% ± 2.33%, while in PDN group rats it was 42.75% ± 1.60%, *p*-value <0.0001). After LQ and MCC950 treatment, the expression of AQP4 increased, and its polar distribution was restored (comparing with group PDN, the “polarization” ratio of AQP4 in LQ group rats was 64.93% ± 2.66%, *p*-value <0.0001; in group MCC950, it was 76.28% ± 1.42%, *p*-value <0.0001). These results indicate that LQ and MCC950 treatments can effectively reduce the number of activated astrocytes and restore the polar distribution of AQP4 around astrocytes in PDN rats.

### 3.4 LQ and MC950 can restore the polarity distribution of AQP4 around blood vessels

**FIGURE 5:**
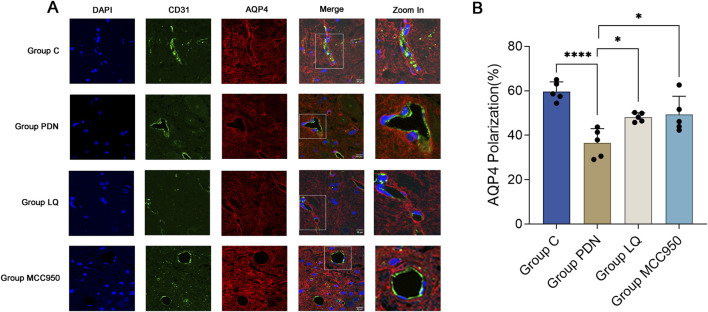
Immunofluorescence results of AQP4 and CD31. **(A)** Immunofluorescent staining was performed on the lumbar enlargement segments of the spinal cord in the C, PDN, LQ, and MCC950 groups of rats. Blood vessels were marked with CD31 and identified by green fluorescence. AQP4 protein was identified with red fluorescence, and cell nuclei were counterstained with DAPI (blue). Enlarged sections detailed the polarity reversal of AQP4 protein. Scale bar = 50 µm. **(B)** Quantitative analysis of fluorescence polarization of AQP4. Refer to Figure 1 for the meaning of the grouping abbreviation. Values were presented as mean ± SEM. Compared to the group PDN, ^*^ *p* < 0.05, ^****^ *p* < 0.0001.

We used vascular endothelial cell specific marker CD31 to mark blood vessels, in order to reflect the localization changes of AQP4. As shown in Figure 5A, perivascular AQP4 localization of PDN rats changed compared with group C, fluorescence localization showed that its continuity was interrupted, and quantitative analysis indicated decreased AQP4 expression, that is, polarity disappeared (Figure 5B, AQP4 “polarization” of rats in Group C, 59.73% ± 1.72% *versus* 36.52% ± 2.59% of rats in Group PDN, ****p < 0.0001). After treatment with LQ and MCC950, AQP4 expression increased and polarity recovered (compared with group PDN, AQP4 “polarization” of rats in Group LQ, 48.10% ± 0.80%, *p < 0.05; Group MCC950, 49.33% ± 3.29%, *p < 0.05).

### 3.5 The effect of LQ in PDN is similar to MCC950, both of which can inhibit the expression of NLRP3 and MMP9 and increase the expression of APQ4 in the glymphatic system

**FIGURE 6:**
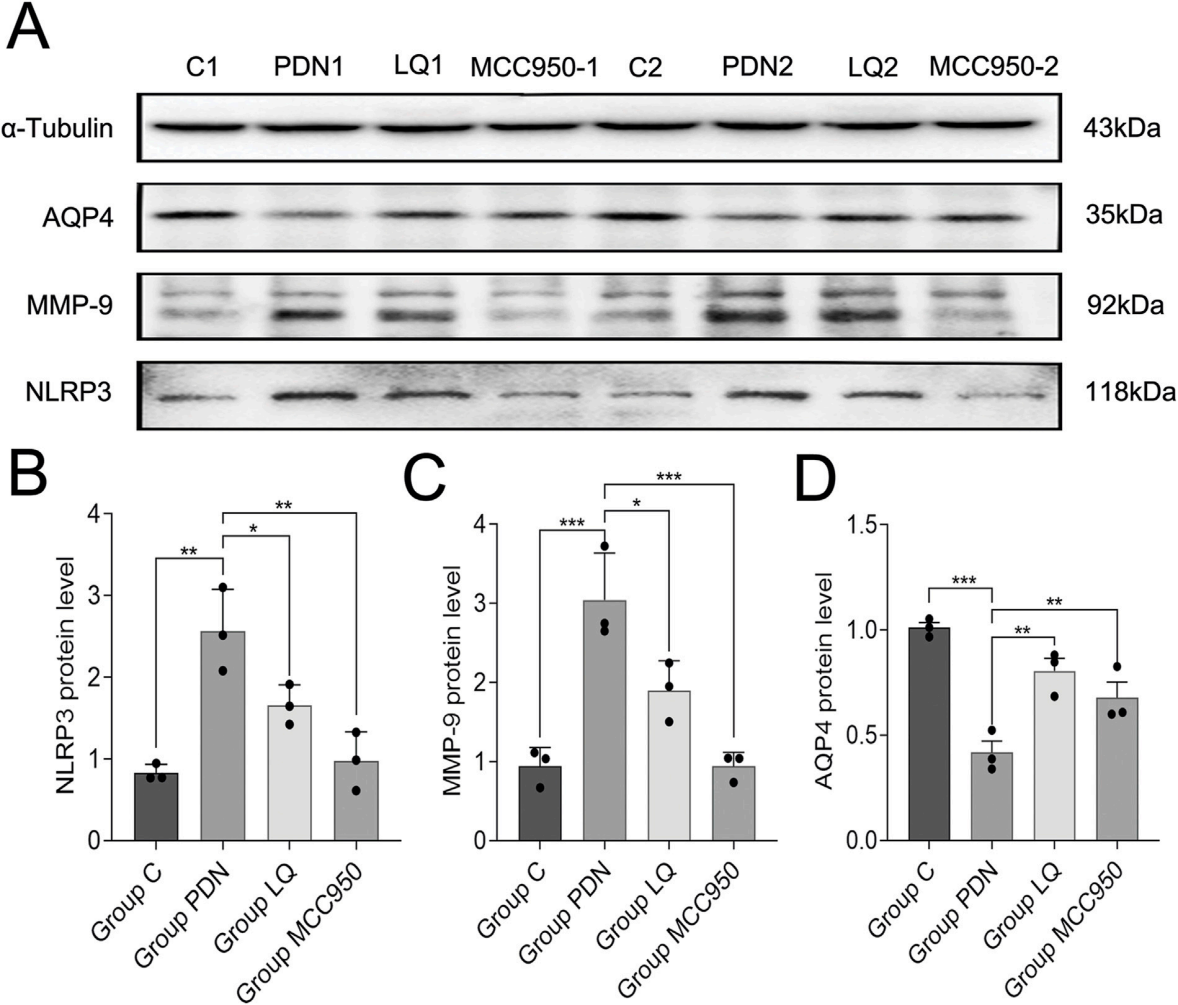
Western blot results. **(A)** Western blot analysis of NLRP3, MMP-9 and AQP4 protein expression in Group C, Group PDN, Group LQ and Group MCC950. **(B)** Relative expression of NLRP3 protein in each group. **(C)** Relative expression of MMP-9 protein in each group. **(D)** Relative expression of AQP4 protein in each group. Refer to Figure 1 for the meaning of the grouping abbreviation. Values were presented as mean ± SEM. Compared with values in Group PDN, ^∗^ *p* < 0.05, ^**^ *p* < 0.01 and ^***^ *p* < 0.001.

The above results still cannot elucidate the molecular mechanisms by which LQ regulates the polarization distribution of AQP4. MMP-9 is associated with the localization and expression of AQP4, while NLRP3 is involved in the regulation of pain occurrence and development and can participate in the modulation of MMP-9 expression. Therefore, we examined the changes in NLRP3, MMP-9, and AQP4 in the spinal cord of PDN rats (Figure 6A–D). Western blot analysis revealed that compared to the group C, the expression of NLRP3 increased in the spinal cord of PDN rats (group C: 0.83 ± 0.05, group PDN: 2.57 ± 0.24, *p* ˂ 0.01), MMP-9 expression increased (group C: 0.94 ± 0.11, group PDN: 3.04 ± 0.28, *p* ˂ 0.001), and AQP4 expression decreased (group C: 1.01 ± 0.02, group PDN: 0.42 ± 0.05, *p* ˂ 0.001). In contrast, after LQ treatment, compared to the group PDN, NLRP3 expression decreased (1.66 ± 0.12, *p* ˂ 0.05), MMP-9 expression decreased (1.90 ± 0.18, *p* ˂ 0.05), and AQP4 expression significantly increased (0.81 ± 0.05, *p* ˂ 0.01). Similarly, the NLRP3 inhibitor MCC950 had effects in PDN similar to LQ. Compared to the group PDN, rats in the group MCC950 exhibited reduced expression of NLRP3 (0.98 ± 0.17, *p* ˂ 0.01), decreased MMP-9 expression (0.94 ± 0.08, *p* ˂ 0.001), and increased AQP4 expression (0.68 ± 0.06, *p* ˂ 0.01) in the spinal cord. These results indicate that LQ can prevent the upregulation of the NLRP3-MMP-9 signaling pathway induced by PDN.

## 4 Discussion

In this investigation, our observations revealed the following key findings: 1) In PDN rats, the clearance function of the spinal glymphatic system is impaired. Experimental findings indicate that astrocytes in the lumbar enlargement of the spinal cord are activated, with upregulation of NLRP3 and MMP-9 expression. Simultaneously, AQP4 expression is reduced, and its polarity distribution is altered. 2) Both liquiritin and MCC950 effectively mitigated PDN symptoms without exerting an impact on blood glucose levels. 3) Post liquiritin treatment, mechanical allodynia decreased in PDN rats, the spinal glymphatic system’s clearance rate increased, NLRP3 and MMP-9 expression decreased, the number of activated astrocytes reduced, and AQP4 expression increased, and the polarity of AQP4 protein was restored. 4) The NLRP3 inflammasome inhibitor MCC950 also facilitated the reduction of MMP-9 and the increase of AQP4, thus restoring AQP4 protein polarity and alleviating PDN, displaying a similar effect to liquiritin. In summary, our contention is that alterations in AQP4 expression level and localization play a pivotal role in the progression of PDN. Liquiritin may alleviate PDN by inhibiting NLRP3, subsequently reducing MMP-9 expression, and restoring the polarity of AQP4.

The literature suggests that estrogen influences blood glucose levels in female SD rats, leading to greater instability and a reduction in glucose levels following STZ injection (Paschou and Papanas, 2019; Ropero et al., 2008). Consequently, this study predominantly utilized male SD rats as subjects. PDN is more commonly associated with type 2 diabetes (Amutha et al., 2021; An et al., 2021). Therefore, we induced type 2 diabetes in rats by combining a high-fat, high-sugar diet with low-dose STZ injected intraperitoneally. (Furman, 2021). Through this method, we achieved a probability of approximately 50% in obtaining rats with PDN, and these PDN rats consistently exhibited stable peripheral neuropathic pain.

The glymphatic system resembles lymphatic vessels within the central nervous system, responsible for clearing soluble waste proteins and metabolic byproducts. Similarly, the spinal cord glymphatic system shares similar structure and functions. The foundational structure of the glymphatic system is the perivascular spaces (PVS) surrounding blood vessels. These PVS consist of arteries or veins, astrocytes, and AQP4 proteins densely distributed on the astrocytic end-feet, forming a potential pathway through which lymphatic fluid can flow (Troili et al., 2020). The size of the PVS space influences the speed of lymphatic fluid flow, which can be achieved by adjusting the end-feet of the astrocytes and the vascular system within the PVS (Schain et al., 2017). An increasing body of research suggests that dysfunction of the glymphatic system is associated with various diseases. In mouse models of traumatic brain injury, early impairment of glymphatic function has been observed (Iliff et al., 2014). Slowed interstitial solute clearance has also been noted in type II diabetes, with the degree of glymphatic system damage correlating with cognitive decline (Jiang et al., 2017). Additionally, dysfunction of the glymphatic system has been implicated in diseases such as Alzheimer’s disease, Parkinson’s disease, and ischemic stroke (Liu et al., 2024; Buccellato et al., 2022). Due to its high sensitivity and non-invasive nature, MRI has been used for over a decade to assess the clearance function of the rat glymphatic system. (Iliff et al., 2013a). In this study, rats were first anesthetized, followed by lumbar puncture at the L4-L5 level. Subsequently, the contrast agent Gd-DTPA was slowly injected into the subarachnoid space within five minutes. The contrast agent initially appeared as a discontinuous distribution on the pia mater’s surface, predominantly accumulating in the anterior median fissure and posterior median sulcus. It then progressively permeated into the anterior and posterior lateral sulci on both sides of the spinal gray matter. This is similar to the results of previous studies (Wei et al., 2017). We hypothesize that the contrast agent may traverse into the gray matter via the interstices around the anterior and posterior spinal arteries or the penetrating arteries of the pia mater. However, the limited resolution of the 3.0T MRI impeded precise observation of the contrast agent’s flow path. Future studies, therefore, intend to employ two-photon microscopy to more accurately trace the flow pathways within the rat spinal glymphatic system.

Before the injection of Gd-DTPA, the initial SI values did not significantly differ across the four groups of rats. In addition, while there was no statistically significant difference in the PEAK TIME among the four groups, the duration needed to reach peak SI values was notably longer in the PDN group relative to the other three groups. This phenomenon may be attributed to the accumulation of advanced glycation end products, release of inflammatory factors causing perivascular space enlargement (Kress et al., 2014). Increasing the sample size in future studies may lead to statistically significant results. On the other hand, compared to the other three groups, the SIPH of the PDN group was decreased. This suggests a reduced clearance rate of the spinal glymphatic system in PDN rats, which collectively could exacerbate the condition of PDN in the rat population. Subsequent to treatment with LQ and MCC950, metabolic function was reinstated in PDN rats, suggesting that both agents effectively enhance the clearance capability of the spinal glymphatic system in PDN rats against the contrast agent. In addition, an increase in PWT was observed in PDN rats, indicative of reduced pain symptoms; however, no significant alteration in blood glucose levels was noted. Consequently, it is hypothesized that the primary mechanism of liquiritin’s therapeutic effect in PDN is through the restoration of the spinal glymphatic system’s clearance function rather than the control of blood glucose.

AQP4 is well-established as predominantly expressed in the central nervous system, particularly concentrated in the foot processes of astrocytes within this system (Papadopoulos and Verkman, 2013; Nielsen et al., 1997). Its functions extend beyond maintaining water and electrolyte balance to include a crucial role in clearing metabolic waste from the brain (Iliff et al., 2012). Numerous studies highlight associations between alterations in AQP4 expression or localization and various central nervous system diseases, such as ischemic stroke (Manley et al., 2000), epilepsy (Hubbard et al., 2016), neuropathic pain (Xian et al., 2021), and glioblastoma (Wang R. et al., 2023). Prior research has demonstrated changes in the polarity of AQP4 in the spinal glymphatic system of rats with PDN (Wang G. Q. et al., 2022). In our investigation, we observed that liquiritin treatment could reinstate the polarity inversion of AQP4 in the spinal glymphatic system of PDN rats. This observation suggests a significant role for changes in AQP4 localization in the progression of PDN, and the restoration of polarized AQP4 expression in astrocyte foot processes may emerge as a novel strategy for PDN treatment. Notably, in the brains of rats with Parkinson’s disease and cerebral ischemia, MMP-9 not only co-localizes with AQP4 but also an increase in MMP-9 expression results in altered AQP4 polarity, (i.e., “depolarization”), MMP-9 disturbs aquaporin-4 polarization by cleaving β-dystroglycan (Si et al., 2024; Yan et al., 2016). Correspondingly, our findings in PDN rats demonstrate an increase in MMP-9 at the lumbar enlargement of the spinal cord, accompanied by reduced AQP4 expression, elevated β-DG protein expression and disrupted polarized distribution of AQP4 (Li et al., 2024). We suggest that during PDN progression, MMP-9 similarly influences the polarized distribution of AQP4. Additionally, our study indicates that treating PDN rats with the NLRP3 inflammasome inhibitor MCC950 yields effects akin to those of liquiritin, suggesting NLRP3’s involvement in regulating MMP-9 and AQP4 expression in PDN. Thus, in PDN pathology, NLRP3, as upstream regulators of AQP4 and MMP-9, plays a crucial role in modulating the localization of AQP4.

In this study, a decrease in AQP4 expression was observed in the spinal glymphatic system of PDN rats, conflicting with prior research findings (Wang F. X. et al., 2022; Xu et al., 2023). Several potential explanations are considered. Firstly, AQP4 expression exhibits bidirectional changes in neurological disorders. For instance, in cerebral ischemic stroke and brain edema, reducing AQP4 activity can mitigate symptoms (Papadopoulos and Verkman, 2007; Tang and Yang, 2016). Conversely, in edema of vascular origin, enhancing AQP4 expression and activity facilitates the clearance of excess water, thereby alleviating symptoms (Verkman et al., 2017). This implies that AQP4 expression levels fluctuate under different pathological conditions. Secondly, research suggests that AQP4, when presented in reactive astrocytes, displays a depolarized distribution. This may originate from either a decrease in AQP4 expression in end-feet or an increase in AQP4 expression in the parenchymal membrane (Wolburg et al., 2011). Such observations indicate the dynamic shifts in AQP4 polarity distribution. It is widely understood that with the advancement of diabetes, a concurrent thickening of both microvessels and parenchymal membranes occurs (Ljubimov et al., 1996). This augmentation may potentially impact the expression of AQP4 on the parenchymal membrane. As such, contradictory results may arise from detecting at different time points due to these dynamic changes. Lastly, the polarity of AQP4 is notably influenced by the ratio of its isoforms M1 and M23 (Zhang J. et al., 2017). In the glymphatic system, tetramers of AQP4 form supramolecular structures, with the M23 isoform comprising the core of these structures, while the M1 isoform is distributed peripherally (Papadopoulos and Verkman, 2013). Following hypoglycemia, an increase in the AQP4-M1/M23 ratio may eventuate, possibly resulting in diminished AQP4 polarity (Deng et al., 2014). *In vitro* studies have demonstrated that at lower concentrations, β-amyloid (1-42) augments AQP4 expression in mouse cortical astrocytes, whereas at higher concentrations, it has the opposite effect (Yang et al., 2012). This observation implies that variables such as blood sugar levels and drug dosage may influence AQP4 expression. In summary, the determinants of AQP4 expression changes are multifaceted, and their presence in PDN still necessitates further investigation. However, it is clear that irrespective of the direction of change in AQP4 expression, the alteration in its polarity is a crucial factor in the onset and progression of PDN.

In the course of this study, sevoflurane was utilized consistently throughout the entire MRI scanning process to ensure consistent rat anesthesia, with scanning intervals permitting the rats to recover autonomously and hydrate. While some research suggest that regular water intake does not alter the total brain water content (Meyers et al., 2016). As such, we believe that normal water consumption does not impact the total cerebrospinal fluid (CSF) volume and does not exerting influence on the spinal glymphatic system. It is noteworthy that studies have indicated sevoflurane may elevate the expression of AQP4 and reduce the expression of NLRP3 and MMP-9 (Gao et al., 2019; Shang et al., 2023; Zheng et al., 2022; Li et al., 2014). However, within our experimental framework, all rats were anesthetized using consistent oxygen flow rates and sevoflurane concentrations for induction and maintenance of anesthesia. Experimental procedures during anesthesia (intrathecal injection, MRI scanning) were conducted within standardized time limits. Therefore, all rats involved in the MRI scans received similar doses and durations of sevoflurane anesthesia. In addition, compared to other anesthetics, sevoflurane acts quickly and allows rapid recovery, enabling rats to awaken and self-administer water and food within a short period. For diabetic rats, this shorter recovery time with the drug ensures maximum tolerance throughout the entire experimental process. Consequently, we hold the view that the effect of sevoflurane on AQP4, NLRP3, and MMP-9 is negligible. Nonetheless, further exploration is required to ascertain the potential influence of sevoflurane anesthesia on the expression levels of NLRP3, MMP-9, and AQP4 in the glymphatic system of the spinal cord in rats with PDN.

Liquiritin, a flavonoid derivative extracted from licorice. It inhibits the activation of the NLRP3 inflammasome, exhibiting antidepressant and neuroprotective effects, and significantly reduces MMP-9 expression in myocardial infarction models (Han et al., 2022). Owing to its anti-inflammatory, anti-injury, and analgesic efficacy across various pain models, liquiritin is increasingly recognized as a promising therapeutic for chronic pain management. Research in a rat model of bone cancer pain revealed that liquiritin mitigates pain by impeding the spinal cord astrocyte CXCL1 and neuronal CXCR2 pathways (Ni et al., 2020). In summary, liquiritin can alleviate pathological pain through multiple pathways and regulate the expression of MMP-9. Combined with the results of this experiment, after treatment with liquiritin, PDN rats showed increased PWT and enhanced clearance function of the spinal glymphatic system. The therapeutic effect on PDN is achieved through the inhibition of NLRP3, thereby reducing the expression of MMP-9 and restoring the polarity of AQP4.

The current study does present certain limitations. Primarily, many studies establish that a mixture of low-dose STZ intraperitoneal injection alongside a diet high in fats and sugars effectively constructs a stable Type 2 diabetes model (Islam and Loots du, 2009; Furman, 2021; Liu et al., 2021). Nonetheless, due to restrictions inherent in our experimental environment, we were unable to measure insulin resistance, a significant limitation of our study. Second, while existing literature does suggest a possible control of AQP4 expression by MMP-9 (Si et al., 2024; Yan et al., 2016), in this work we did not apply MMP-9 inhibitors and AQP4 inhibitors to further substantiate such a relationship between MMP-9 and AQP4 in rats with PDN. However, in previous studies, we found that inhibiting MMP-9 expression in PDN rats can restore the polarity of AQP4 protein in the spinal glymphatic system. MMP-9 regulates the expression and distribution of AQP4 by cleaving β-Dystroglycan (Li et al., 2024). Furthermore, our data suggest that inhibition of NLRP3 expression reduces MMP-9 activity. These findings support the hypothesis that liquiritin may improve PDN by inhibiting NLRP3 and thereby regulating MMP-9 and AQP4. Finally, we did not further elaborate on the impact of pericytes on the localization of AQP4. Pericytes, situated on the periphery of vascular endothelial cells, play a crucial role not only in maintaining vascular homeostasis but also in modulating the local microenvironment to influence the function of neurons and astrocytes (Hellström et al., 2001). Specifically, pericytes regulate the polarized distribution of AQP4 in the foot processes of astrocytes (Gundersen et al., 2014). Moreover, pericytes may modulate the function of the glymphatic system through various mechanisms, thereby playing a significant role in central nervous system diseases (Sweeney et al., 2016). For instance, in spinal cord injury models, the upregulation of Ang1 in pericytes not only inhibits inflammation and apoptosis but also suppresses the upregulation of AQP4, thus protecting microvessels (Jing et al., 2014). Additionally, glial cell adhesion proteins maintain the integrity of the blood-brain barrier by regulating the differentiation of pericytes (Yao et al., 2014). Therefore, we hypothesize that in diabetic neuropathic pain, the inflammatory response and changes in intracellular signaling pathways in pericytes may affect the expression levels and distribution of AQP4, subsequently impacting the homeostasis of the glymphatic system and the clearance rate of metabolic products. Future research should thoroughly investigate the interactions between pericytes and AQP4 at the cellular and subcellular levels, and reveal how these interactions evolve with the progression of diabetic neuropathic pain. This will help deepen our understanding of the role of pericytes in the pathogenesis of neurological diseases and provide a theoretical basis for the development of targeted therapeutic strategies.

This study effectively established a Type 2 diabetes model using a high-fat, high-sugar diet combined with low-dose STZ intraperitoneal injection and subsequently developed a stable PDN rat model. Key findings include a reduction in PWT in PDN rats, paralleled by impaired clearance in the spinal glymphatic system for contrast agents. Localization analysis indicated changes in the polarity of AQP4 around blood vessels and activated astrocytes. Additionally, quantitative assessments revealed an upsurge in NLRP3 and MMP-9 expression, an increase in activated astrocytes, and a reduction in AQP4 expression. Following treatment with liquiritin and MCC950, Upon administering liquiritin and MCC950, there was a notable enhancement in PWT in PDN rats, improved clearance ability of the spinal glymphatic system for contrast agents, restoration of AQP4 polarity around blood vessels and activated astrocytes, diminished NLRP3 and MMP-9 expression, a decrease in activated astrocytes, and an elevation in AQP4 expression. It is noteworthy that this study is the first to elucidate the roles of NLRP3 and MMP-9 in regulating the expression and localization of AQP4 within the spinal cord glymphatic system of rats with PDN. The results indicate that liquiritin may promote the restoration of AQP4 polarity by inhibiting NLRP3 and MMP-9, thereby enhancing the clearance functions of the spinal cord glymphatic system in PDN rats, alleviating the progression of PDN, and offering a novel approach for its prevention.

## Data Availability

The original contributions presented in the study are included in the article/Supplementary Material, further inquiries can be directed to the corresponding author.

## Abbreviations

AQP4, aquaporin-4; CNS, central nervous system; Gd-DTPA, gadopentetic acid; GFAP, glial fibrillary acidic protein; LQ, liquiritin; MMP-9, matrix metalloproteinase-9; MRI SI, MRI signal intensity; MRI, magnetic resonance imaging; NLRP3, The NOD-, LRR- and pyrin domain-containing protein 3; PDN, painful diabetic neuropathy; PWT, paw withdrawal threshold; STZ, streptozotocin; TCAs, tricyclic antidepressants; TNF-α, tumor necrosis factor-alpha.

## References

[B1] Abbott C. A. Malik R. A. Van Ross E. R. Kulkarni J. Boulton A. J. (2011). Prevalence and characteristics of painful diabetic neuropathy in a large community-based diabetic population in the U.K. Diabetes Care 34 (10), 2220–2224. 10.2337/dc11-1108 21852677 PMC3177727

[B2] Aman U. Subhan F. Shahid M. Akbar S. Ahmad N. Ali G. (2016). Passiflora incarnata attenuation of neuropathic allodynia and vulvodynia apropos GABA-ergic and opioidergic antinociceptive and behavioural mechanisms. BMC Complement. Altern. Med. 16, 77. 10.1186/s12906-016-1048-6 26912265 PMC4765057

[B3] Amutha A. Ranjit U. Anjana R. M. Shanthi R. C. Rajalakshmi R. Venkatesan U. (2021). Clinical profile and incidence of microvascular complications of childhood and adolescent onset type 1 and type 2 diabetes seen at a tertiary diabetes center in India. Pediatr. Diabetes 22 (1), 67–74. 10.1111/pedi.13033 32333449

[B4] An J. Nichols G. A. Qian L. Munis M. A. Harrison T. N. Li Z. (2021). Prevalence and incidence of microvascular and macrovascular complications over 15 years among patients with incident type 2 diabetes. BMJ Open Diabetes Res. Care 9 (1), e001847. 10.1136/bmjdrc-2020-001847 PMC7783518 33397671

[B5] Aronson R. Chu L. Joseph N. Brown R. (2021). Prevalence and risk evaluation of diabetic complications of the foot among adults with type 1 and type 2 diabetes in a large Canadian population (PEDAL study). Can. J. Diabetes 45 (7), 588–593. 10.1016/j.jcjd.2020.11.011 33582042

[B6] Buccellato F. R. D'anca M. Serpente M. Arighi A. Galimberti D. (2022). The role of glymphatic system in Alzheimer's and Parkinson's disease pathogenesis. Biomedicines 10 (9), 2261. 10.3390/biomedicines10092261 36140362 PMC9496080

[B7] Chaplan S. R. Bach F. W. Pogrel J. W. Chung J. M. Yaksh T. L. (1994). Quantitative assessment of tactile allodynia in the rat paw. J. Neurosci. Methods 53 (1), 55–63. 10.1016/0165-0270(94)90144-9 7990513

[B8] Chen Y. Y. Feng L. M. Xu D. Q. Yue S. J. Fu R. J. Zhang M. M. (2022). Combination of paeoniflorin and liquiritin alleviates neuropathic pain by lipid metabolism and calcium signaling coordination. Front. Pharmacol. 13, 944386. 10.3389/fphar.2022.944386 36160378 PMC9489943

[B9] Cheng M. Zhang J. Yang L. Shen S. Li P. Yao S. (2021). Recent advances in chemical analysis of licorice (Gan-Cao). Fitoterapia 149, 104803. 10.1016/j.fitote.2020.104803 33309652

[B10] Dai J. Lin W. Zheng M. Liu Q. He B. Luo C. (2017). Alterations in AQP4 expression and polarization in the course of motor neuron degeneration in SOD1G93A mice. Mol. Med. Rep. 16 (2), 1739–1746. 10.3892/mmr.2017.6786 28627708 PMC5562093

[B11] Das A. Wallace G. C. T. Holmes C. Mcdowell M. L. Smith J. A. Marshall J. D. (2012). Hippocampal tissue of patients with refractory temporal lobe epilepsy is associated with astrocyte activation, inflammation, and altered expression of channels and receptors. Neuroscience 220, 237–246. 10.1016/j.neuroscience.2012.06.002 22698689 PMC3412889

[B12] Deng J. Zhao F. Yu X. Zhao Y. Li D. Shi H. (2014). Expression of aquaporin 4 and breakdown of the blood-brain barrier after hypoglycemia-induced brain edema in rats. PLoS One 9 (9), e107022. 10.1371/journal.pone.0107022 25264602 PMC4180270

[B13] Furman B. L. (2021). Streptozotocin-induced diabetic models in mice and rats. Curr. Protoc. 1 (4), e78. 10.1002/cpz1.78 33905609

[B14] Gao X. Ming J. Liu S. Lai B. Fang F. Cang J. (2019). Sevoflurane enhanced the clearance of Aβ1-40 in hippocampus under surgery via up-regulating AQP-4 expression in astrocyte. Life Sci. 221, 143–151. 10.1016/j.lfs.2019.02.024 30763576

[B15] Gomolka R. S. Hablitz L. M. Mestre H. Giannetto M. Du T. Hauglund N. L. (2023). Loss of aquaporin-4 results in glymphatic system dysfunction via brain-wide interstitial fluid stagnation. Elife 12, e82232. 10.7554/eLife.82232 36757363 PMC9995113

[B16] Griebeler M. L. Morey-Vargas O. L. Brito J. P. Tsapas A. Wang Z. Carranza Leon B. G. (2014). Pharmacologic interventions for painful diabetic neuropathy: an umbrella systematic review and comparative effectiveness network meta-analysis. Ann. Intern Med. 161 (9), 639–649. 10.7326/m14-0511 25364885

[B17] Gundersen G. A. Vindedal G. F. Skare O. Nagelhus E. A. (2014). Evidence that pericytes regulate aquaporin-4 polarization in mouse cortical astrocytes. Brain Struct. Funct. 219 (6), 2181–2186. 10.1007/s00429-013-0629-0 23982198 PMC4223569

[B18] Gylfadottir S. S. Christensen D. H. Nicolaisen S. K. Andersen H. Callaghan B. C. Itani M. (2020). Diabetic polyneuropathy and pain, prevalence, and patient characteristics: a cross-sectional questionnaire study of 5,514 patients with recently diagnosed type 2 diabetes. Pain 161 (3), 574–583. 10.1097/j.pain.0000000000001744 31693539 PMC7017941

[B19] Haj-Yasein N. N. Jensen V. Østby I. Omholt S. W. Voipio J. Kaila K. (2012). Aquaporin-4 regulates extracellular space volume dynamics during high-frequency synaptic stimulation: a gene deletion study in mouse hippocampus. Glia 60 (6), 867–874. 10.1002/glia.22319 22419561

[B20] Han X. Yang Y. Zhang M. Li L. Xue Y. Jia Q. (2022). Liquiritin protects against cardiac fibrosis after myocardial infarction by inhibiting CCL5 expression and the NF-κB signaling pathway. Drug Des. Devel Ther. 16, 4111–4125. 10.2147/dddt.S386805 PMC9724582 36483459

[B21] Hellström M. Gerhardt H. Kalén M. Li X. Eriksson U. Wolburg H. (2001). Lack of pericytes leads to endothelial hyperplasia and abnormal vascular morphogenesis. J. Cell Biol. 153 (3), 543–553. 10.1083/jcb.153.3.543 11331305 PMC2190573

[B22] Hubbard J. A. Szu J. I. Yonan J. M. Binder D. K. (2016). Regulation of astrocyte glutamate transporter-1 (GLT1) and aquaporin-4 (AQP4) expression in a model of epilepsy. Exp. Neurol. 283 (Pt A), 85–96. 10.1016/j.expneurol.2016.05.003 27155358 PMC4992641

[B23] Iliff J. J. Chen M. J. Plog B. A. Zeppenfeld D. M. Soltero M. Yang L. (2014). Impairment of glymphatic pathway function promotes tau pathology after traumatic brain injury. J. Neurosci. 34 (49), 16180–16193. 10.1523/jneurosci.3020-14.2014 25471560 PMC4252540

[B24] Iliff J. J. Lee H. Yu M. Feng T. Logan J. Nedergaard M. (2013a). Brain-wide pathway for waste clearance captured by contrast-enhanced MRI. J. Clin. Invest. 123 (3), 1299–1309. 10.1172/jci67677 23434588 PMC3582150

[B25] Iliff J. J. Wang M. Liao Y. Plogg B. A. Peng W. Gundersen G. A. (2012). A paravascular pathway facilitates CSF flow through the brain parenchyma and the clearance of interstitial solutes, including amyloid β. Sci. Transl. Med. 4 (147), 147ra111. 10.1126/scitranslmed.3003748 PMC3551275 22896675

[B26] Iliff J. J. Wang M. Zeppenfeld D. M. Venkataraman A. Plog B. A. Liao Y. (2013b). Cerebral arterial pulsation drives paravascular CSF-interstitial fluid exchange in the murine brain. J. Neurosci. 33 (46), 18190–18199. 10.1523/jneurosci.1592-13.2013 24227727 PMC3866416

[B27] Islam M. S. Loots Du T. (2009). Experimental rodent models of type 2 diabetes: a review. Methods Find. Exp. Clin. Pharmacol. 31 (4), 249–261. 10.1358/mf.2009.31.4.1362513 19557203

[B28] Jessen N. A. Munk A. S. Lundgaard I. Nedergaard M. (2015). The glymphatic system: a beginner's guide. Neurochem. Res. 40 (12), 2583–2599. 10.1007/s11064-015-1581-6 25947369 PMC4636982

[B29] Jiang Q. Zhang L. Ding G. Davoodi-Bojd E. Li Q. Li L. (2017). Impairment of the glymphatic system after diabetes. J. Cereb. Blood Flow. Metab. 37 (4), 1326–1337. 10.1177/0271678x16654702 27306755 PMC5453454

[B30] Jing Y. Wu Q. Yuan X. Li B. Liu M. Zhang X. (2014). Microvascular protective role of pericytes in melatonin-treated spinal cord injury in the C57BL/6 mice. Chin. Med. J. Engl. 127 (15), 2808–2813. 25146619

[B31] Kress B. T. Iliff J. J. Xia M. Wang M. Wei H. S. Zeppenfeld D. (2014). Impairment of paravascular clearance pathways in the aging brain. Ann. Neurol. 76 (6), 845–861. 10.1002/ana.24271 25204284 PMC4245362

[B32] Li J. Jia S. Song Y. Xu W. Lin J. (2024). Ginkgolide B can alleviate spinal cord glymphatic system dysfunction and provide neuroprotection in painful diabetic neuropathy rats by inhibiting matrix metalloproteinase-9. Neuropharmacology 250, 109907. 10.1016/j.neuropharm.2024.109907 38492884

[B33] Li X. Q. Cai L. M. Liu J. Ma Y. L. Kong Y. H. Li H. (2018). Liquiritin suppresses UVB-induced skin injury through prevention of inflammation, oxidative stress and apoptosis through the TLR4/MyD88/NF-κB and MAPK/caspase signaling pathways. Int. J. Mol. Med. 42 (3), 1445–1459. 10.3892/ijmm.2018.3720 29901082 PMC6089709

[B34] Li X. Q. Cao X. Z. Wang J. Fang B. Tan W. F. Ma H. (2014). Sevoflurane preconditioning ameliorates neuronal deficits by inhibiting microglial MMP-9 expression after spinal cord ischemia/reperfusion in rats. Mol. Brain 7, 69. 10.1186/s13041-014-0069-7 25186151 PMC4161899

[B35] Liu S. Lam M. A. Sial A. Hemley S. J. Bilston L. E. Stoodley M. A. (2018). Fluid outflow in the rat spinal cord: the role of perivascular and paravascular pathways. Fluids Barriers CNS 15 (1), 13. 10.1186/s12987-018-0098-1 29704892 PMC5924677

[B36] Liu S. Ma L. Ren X. Zhang W. Shi D. Huo Y. (2021). A new mouse model of type 2 diabetes mellitus established through combination of high-fat diet, streptozotocin and glucocorticoid. Life Sci. 286, 120062. 10.1016/j.lfs.2021.120062 34673117

[B37] Liu S. Sun X. Ren Q. Chen Y. Dai T. Yang Y. (2024). Glymphatic dysfunction in patients with early-stage amyotrophic lateral sclerosis. Brain 147 (1), 100–108. 10.1093/brain/awad274 37584389

[B38] Ljubimov A. V. Burgeson R. E. Butkowski R. J. Couchman J. R. Zardi L. Ninomiya Y. (1996). Basement membrane abnormalities in human eyes with diabetic retinopathy. J. Histochem Cytochem 44 (12), 1469–1479. 10.1177/44.12.8985139 8985139

[B39] Luo Y. Yan W. Zhou Z. Liu B. Wang Z. Chen J. (2019). Elevated levels of NLRP3 in cerebrospinal fluid of patients with autoimmune GFAP astrocytopathy. Front. Neurol. 10, 1019. 10.3389/fneur.2019.01019 31681133 PMC6812676

[B40] Manley G. T. Fujimura M. Ma T. Noshita N. Filiz F. Bollen A. W. (2000). Aquaporin-4 deletion in mice reduces brain edema after acute water intoxication and ischemic stroke. Nat. Med. 6 (2), 159–163. 10.1038/72256 10655103

[B41] Mestre H. Hablitz L. M. Xavier A. L. Feng W. Zou W. Pu T. (2018). Aquaporin-4-dependent glymphatic solute transport in the rodent brain. Elife 7, e40070. 10.7554/eLife.40070 30561329 PMC6307855

[B42] Meyers S. M. Tam R. Lee J. S. Kolind S. H. Vavasour I. M. Mackie E. (2016). Does hydration status affect MRI measures of brain volume or water content? J. Magn. Reson Imaging 44 (2), 296–304. 10.1002/jmri.25168 26825048

[B43] Ni H. Xu M. Xie K. Fei Y. Deng H. He Q. (2020). Liquiritin alleviates pain through inhibiting CXCL1/CXCR2 signaling pathway in bone cancer pain rat. Front. Pharmacol. 11, 436. 10.3389/fphar.2020.00436 32390832 PMC7193085

[B44] Nielsen S. Nagelhus E. A. Amiry-Moghaddam M. Bourque C. Agre P. Ottersen O. P. (1997). Specialized membrane domains for water transport in glial cells: high-resolution immunogold cytochemistry of aquaporin-4 in rat brain. J. Neurosci. 17 (1), 171–180. 10.1523/jneurosci.17-01-00171.1997 8987746 PMC6793699

[B45] Nirogi R. Goura V. Shanmuganathan D. Jayarajan P. Abraham R. (2012). Comparison of manual and automated filaments for evaluation of neuropathic pain behavior in rats. J. Pharmacol. Toxicol. Methods 66 (1), 8–13. 10.1016/j.vascn.2012.04.006 22575456

[B46] Papadopoulos M. C. Verkman A. S. (2007). Aquaporin-4 and brain edema. Pediatr. Nephrol. 22 (6), 778–784. 10.1007/s00467-006-0411-0 17347837 PMC6904420

[B47] Papadopoulos M. C. Verkman A. S. (2013). Aquaporin water channels in the nervous system. Nat. Rev. Neurosci. 14 (4), 265–277. 10.1038/nrn3468 23481483 PMC3732112

[B48] Paschou S. A. Papanas N. (2019). Type 2 diabetes mellitus and menopausal hormone therapy: an update. Diabetes Ther. 10 (6), 2313–2320. 10.1007/s13300-019-00695-y 31549295 PMC6848654

[B49] Peng L. Wen L. Shi Q. F. Gao F. Huang B. Meng J. (2020). Scutellarin ameliorates pulmonary fibrosis through inhibiting NF-κB/NLRP3-mediated epithelial-mesenchymal transition and inflammation. Cell Death Dis. 11 (11), 978. 10.1038/s41419-020-03178-2 33188176 PMC7666141

[B50] Ren C. Chen M. Mu G. Peng S. Liu X. Ou C. (2021). NLRP3 inflammasome mediates neurodegeneration in rats with chronic neuropathic pain. Shock 56 (5), 840–849. 10.1097/shk.0000000000001832 34265833

[B51] Ropero A. B. Alonso-Magdalena P. Quesada I. Nadal A. (2008). The role of estrogen receptors in the control of energy and glucose homeostasis. Steroids 73 (9-10), 874–879. 10.1016/j.steroids.2007.12.018 18249429

[B52] Schain A. J. Melo-Carrillo A. Strassman A. M. Burstein R. (2017). Cortical spreading depression closes paravascular space and impairs glymphatic flow: implications for migraine headache. J. Neurosci. 37 (11), 2904–2915. 10.1523/jneurosci.3390-16.2017 28193695 PMC5354333

[B53] Seifert G. Schilling K. Steinhäuser C. (2006). Astrocyte dysfunction in neurological disorders: a molecular perspective. Nat. Rev. Neurosci. 7 (3), 194–206. 10.1038/nrn1870 16495941

[B54] Shang S. Sun F. Zhu Y. Yu J. Yu L. Shao W. (2023). Sevoflurane preconditioning improves neuroinflammation in cerebral ischemia/reperfusion induced rats through ROS-NLRP3 pathway. Neurosci. Lett. 801, 137164. 10.1016/j.neulet.2023.137164 36868396

[B55] Si X. Dai S. Fang Y. Tang J. Wang Z. Li Y. (2024). Matrix metalloproteinase-9 inhibition prevents aquaporin-4 depolarization-mediated glymphatic dysfunction in Parkinson's disease. J. Adv. Res. 56, 125–136. 10.1016/j.jare.2023.03.004 36940850 PMC10834796

[B56] Starobova H. Nadar E. I. Vetter I. (2020). The NLRP3 inflammasome: role and therapeutic potential in pain treatment. Front. Physiol. 11, 1016. 10.3389/fphys.2020.01016 32973552 PMC7468416

[B57] Su X. Wu B. Zhang W. Ji Y. H. Wang Q. Tan Z. Y. (2019). Inhibitory effects of columbianadin on nociceptive behaviors in a neuropathic pain model, and on voltage-gated calcium currents in dorsal root ganglion neurons in mice. Front. Pharmacol. 10, 1522. 10.3389/fphar.2019.01522 31998126 PMC6970200

[B58] Sun H. Saeedi P. Karuranga S. Pinkepank M. Ogurtsova K. Duncan B. B. (2022). IDF Diabetes Atlas: global, regional and country-level diabetes prevalence estimates for 2021 and projections for 2045. Diabetes Res. Clin. Pract. 183, 109119. 10.1016/j.diabres.2021.109119 34879977 PMC11057359

[B59] Sun L. Ma W. Gao W. Xing Y. Chen L. Xia Z. (2019). Propofol directly induces caspase-1-dependent macrophage pyroptosis through the NLRP3-ASC inflammasome. Cell Death Dis. 10 (8), 542. 10.1038/s41419-019-1761-4 31316052 PMC6637184

[B60] Sweeney M. D. Ayyadurai S. Zlokovic B. V. (2016). Pericytes of the neurovascular unit: key functions and signaling pathways. Nat. Neurosci. 19 (6), 771–783. 10.1038/nn.4288 27227366 PMC5745011

[B61] Talbot S. Chahmi E. Dias J. P. Couture R. (2010). Key role for spinal dorsal horn microglial kinin B1 receptor in early diabetic pain neuropathy. J. Neuroinflammation 7 (1), 36. 10.1186/1742-2094-7-36 20587056 PMC2913947

[B62] Tang G. Yang G. Y. (2016). Aquaporin-4: a potential therapeutic target for cerebral edema. Int. J. Mol. Sci. 17 (10), 1413. 10.3390/ijms17101413 27690011 PMC5085613

[B63] Tesfaye S. Sloan G. Petrie J. White D. Bradburn M. Young T. (2022). Optimal pharmacotherapy pathway in adults with diabetic peripheral neuropathic pain: the OPTION-DM RCT. Health Technol. Assess. 26 (39), 1–100. 10.3310/rxuo6757 PMC9589396 36259684

[B64] Tesfaye S. Vileikyte L. Rayman G. Sindrup S. H. Perkins B. A. Baconja M. (2011). Painful diabetic peripheral neuropathy: consensus recommendations on diagnosis, assessment and management. Diabetes Metab. Res. Rev. 27 (7), 629–638. 10.1002/dmrr.1225 21695762

[B65] Thomas A. Miller A. Roughan J. Malik A. Haylor K. Sandersen C. (2016). Efficacy of intrathecal morphine in a model of surgical pain in rats. PLoS One 11 (10), e0163909. 10.1371/journal.pone.0163909 27783629 PMC5082666

[B66] Tokhi A. Ahmed Z. Arif M. Rehman N. U. Sheibani V. Sewell R. D. E. (2023). Effects of 1-methyl-1, 2, 3, 4-tetrahydroisoquinoline on a diabetic neuropathic pain model. Front. Pharmacol. 14, 1128496. 10.3389/fphar.2023.1128496 37033637 PMC10073420

[B67] Troili F. Cipollini V. Moci M. Morena E. Palotai M. Rinaldi V. (2020). Perivascular unit: this must Be the place. The anatomical crossroad between the immune, vascular and nervous system. Front. Neuroanat. 14, 17. 10.3389/fnana.2020.00017 32372921 PMC7177187

[B68] Verkman A. S. Smith A. J. Phuan P. W. Tradtrantip L. Anderson M. O. (2017). The aquaporin-4 water channel as a potential drug target in neurological disorders. Expert Opin. Ther. Targets 21 (12), 1161–1170. 10.1080/14728222.2017.1398236 29072508 PMC6481624

[B69] Wang F. X. Xu C. L. Su C. Li J. Lin J. Y. (2022a). β-Hydroxybutyrate attenuates painful diabetic neuropathy via restoration of the aquaporin-4 polarity in the spinal glymphatic system. Front. Neurosci. 16, 926128. 10.3389/fnins.2022.926128 35898407 PMC9309893

[B70] Wang G. Q. Wang F. X. He Y. N. Lin J. Y. (2022b). Plasticity of the spinal glymphatic system in male SD rats with painful diabetic neuropathy induced by type 2 diabetes mellitus. J. Neurosci. Res. 100 (10), 1908–1920. 10.1002/jnr.25104 35796387 PMC9541551

[B71] Wang H. Chen H. Jin J. Liu Q. Zhong D. Li G. (2020). Inhibition of the NLRP3 inflammasome reduces brain edema and regulates the distribution of aquaporin-4 after cerebral ischaemia-reperfusion. Life Sci. 251, 117638. 10.1016/j.lfs.2020.117638 32251636

[B72] Wang H. Yuan M. Yang E. Chen D. Su A. Wu Z. (2021). Enterovirus 71 infection induced Aquaporin-4 depolarization by increasing matrix metalloproteinase-9 activity. Neurosci. Lett. 759, 136049. 10.1016/j.neulet.2021.136049 34126180

[B73] Wang L. Huang C. Li Z. Hu G. Qi J. Fan Z. (2023a). Liquiritin inhibits MRGPRX2-mediated pseudo-allergy through the PI3K/AKT and PLCγ signaling pathways. Heliyon 9 (2), e13290. 10.1016/j.heliyon.2023.e13290 36816265 PMC9932484

[B74] Wang Q. Zhang K. Weng W. Chen L. Wei C. Bao R. (2022c). Liquiritin-hydroxypropyl-beta-cyclodextrin inclusion complex: preparation, characterization, bioavailability and antitumor activity evaluation. J. Pharm. Sci. 111 (7), 2083–2092. 10.1016/j.xphs.2022.03.021 35367247

[B75] Wang R. Peng L. Xiao Y. Zhou Q. Wang Z. Tang L. (2023b). Single-cell RNA sequencing reveals changes in glioma-associated macrophage polarization and cellular states of malignant gliomas with high AQP4 expression. Cancer Gene Ther. 30 (5), 716–726. 10.1038/s41417-022-00582-y 36599974 PMC10191842

[B76] Wei F. Zhang C. Xue R. Shan L. Gong S. Wang G. (2017). The pathway of subarachnoid CSF moving into the spinal parenchyma and the role of astrocytic aquaporin-4 in this process. Life Sci. 182, 29–40. 10.1016/j.lfs.2017.05.028 28576642

[B77] Wolburg H. Wolburg-Buchholz K. Fallier-Becker P. Noell S. Mack A. F. (2011). Structure and functions of aquaporin-4-based orthogonal arrays of particles. Int. Rev. Cell Mol. Biol. 287, 1–41. 10.1016/b978-0-12-386043-9.00001-3 21414585

[B78] Wolburg-Buchholz K. Mack A. F. Steiner E. Pfeiffer F. Engelhardt B. Wolburg H. (2009). Loss of astrocyte polarity marks blood-brain barrier impairment during experimental autoimmune encephalomyelitis. Acta Neuropathol. 118 (2), 219–233. 10.1007/s00401-009-0558-4 19533155

[B79] Xian S. Ding R. Li M. Chen F. (2021). LncRNA NEAT1/miR-128-3p/AQP4 axis regulating spinal cord injury-induced neuropathic pain progression. J. Neuroimmunol. 351, 577457. 10.1016/j.jneuroim.2020.577457 33373887

[B80] Xu C. Wang F. Su C. Guo X. Li J. Lin J. (2023). Restoration of aquaporin-4 polarization in the spinal glymphatic system by metformin in rats with painful diabetic neuropathy. Neuroreport 34 (3), 190–197. 10.1097/wnr.0000000000001880 36719843 PMC9981323

[B81] Yamaguchi T. Miyamoto T. Shikata E. Yamaguchi I. Shimada K. Yagi K. (2023). Activation of the NLRP3/IL-1β/MMP-9 pathway and intracranial aneurysm rupture associated with the depletion of ERα and Sirt1 in oophorectomized rats. J. Neurosurg. 138 (1), 191–198. 10.3171/2022.4.Jns212945 35594890

[B82] Yan W. Zhao X. Chen H. Zhong D. Jin J. Qin Q. (2016). β-Dystroglycan cleavage by matrix metalloproteinase-2/-9 disturbs aquaporin-4 polarization and influences brain edema in acute cerebral ischemia. Neuroscience 326, 141–157. 10.1016/j.neuroscience.2016.03.055 27038751

[B83] Yang W. Wu Q. Yuan C. Gao J. Xiao M. Gu M. (2012). Aquaporin-4 mediates astrocyte response to β-amyloid. Mol. Cell Neurosci. 49 (4), 406–414. 10.1016/j.mcn.2012.02.002 22365952

[B84] Yang X. Dang X. Zhang X. Zhao S. (2021). Liquiritin reduces lipopolysaccharide-aroused HaCaT cell inflammation damage via regulation of microRNA-31/MyD88. Int. Immunopharmacol. 101 (Pt B), 108283. 10.1016/j.intimp.2021.108283 34731782

[B85] Yao Y. Chen Z. L. Norris E. H. Strickland S. (2014). Astrocytic laminin regulates pericyte differentiation and maintains blood brain barrier integrity. Nat. Commun. 5, 3413. 10.1038/ncomms4413 24583950 PMC3992931

[B86] Yin J. J. He Y. An J. Miao Q. Sui R. X. Wang Q. (2019). Dynamic balance of microglia and astrocytes involved in the remyelinating effect of ginkgolide B. Front. Cell Neurosci. 13, 572. 10.3389/fncel.2019.00572 31969806 PMC6960131

[B87] Zeppenfeld D. M. Simon M. Haswell J. D. D'abreo D. Murchison C. Quinn J. F. (2017). Association of perivascular localization of aquaporin-4 with cognition and alzheimer disease in aging brains. JAMA Neurol. 74 (1), 91–99. 10.1001/jamaneurol.2016.4370 27893874

[B88] Zhang J. Zhan Z. Li X. Xing A. Jiang C. Chen Y. (2017a). Intermittent fasting protects against Alzheimer's disease possible through restoring aquaporin-4 polarity. Front. Mol. Neurosci. 10, 395. 10.3389/fnmol.2017.00395 29238290 PMC5712566

[B89] Zhang M. T. Wang B. Jia Y. N. Liu N. Ma P. S. Gong S. S. (2017b). Neuroprotective effect of liquiritin against neuropathic pain induced by chronic constriction injury of the sciatic nerve in mice. Biomed. Pharmacother. 95, 186–198. 10.1016/j.biopha.2017.07.167 28843150

[B90] Zhang Q. Li Q. Liu S. Zheng H. Ji L. Yi N. (2022). Glucagon-like peptide-1 receptor agonist attenuates diabetic neuropathic pain via inhibition of NOD-like receptor protein 3 inflammasome in brain microglia. Diabetes Res. Clin. Pract. 186, 109806. 10.1016/j.diabres.2022.109806 35240228

[B91] Zhang Q. H. Huang H. Z. Qiu M. Wu Z. F. Xin Z. C. Cai X. F. (2021). Traditional uses, pharmacological effects, and molecular mechanisms of licorice in potential therapy of COVID-19. Front. Pharmacol. 12, 719758. 10.3389/fphar.2021.719758 34899289 PMC8661450

[B92] Zheng F. Wu X. Zhang J. Fu Z. (2022). Sevoflurane suppresses NLRP3 inflammasome-mediated pyroptotic cell death to attenuate lipopolysaccharide-induced acute lung injury through inducing GSK-3β phosphorylation and activation. Int. Immunopharmacol. 109, 108800. 10.1016/j.intimp.2022.108800 35550264

[B93] Zheng R. Huang Y. M. Zhou Q. (2021a). Xueshuantong improves functions of lymphatic ducts and modulates inflammatory responses in Alzheimer's disease mice. Front. Pharmacol. 12, 605814. 10.3389/fphar.2021.605814 34650426 PMC8505705

[B94] Zheng T. Wang Q. Bian F. Zhao Y. Ma W. Zhang Y. (2021b). Salidroside alleviates diabetic neuropathic pain through regulation of the AMPK-NLRP3 inflammasome axis. Toxicol. Appl. Pharmacol. 416, 115468. 10.1016/j.taap.2021.115468 33639149

[B95] Zychowska M. Rojewska E. Kreiner G. Nalepa I. Przewlocka B. Mika J. (2013). Minocycline influences the anti-inflammatory interleukins and enhances the effectiveness of morphine under mice diabetic neuropathy. J. Neuroimmunol. 262 (1-2), 35–45. 10.1016/j.jneuroim.2013.06.005 23870534

