# Supplementary material for: Liquiritin ameliorates painful diabetic neuropathy in SD rats by inhibiting NLRP3-MMP-9-mediated reversal of aquaporin-4 polarity in the glymphatic system
Source: Front Pharmacol. 2024 Sep 4;15:1436146. doi: 10.3389/fphar.2024.1436146 (PMC11408323; doi:10.3389/fphar.2024.1436146)
Supplement: Supplementary file 1 [file DataSheet1.docx]

1. AQP4









1. MMP-9









1. NLRP3









1. α-Tubulin
